# White Matter Tissue Quantification at Low *b*-Values Within Constrained Spherical Deconvolution Framework

**DOI:** 10.3389/fneur.2018.00716

**Published:** 2018-08-28

**Authors:** Alessandro Calamuneri, Alessandro Arrigo, Enricomaria Mormina, Demetrio Milardi, Alberto Cacciola, Gaetana Chillemi, Silvia Marino, Michele Gaeta, Angelo Quartarone

**Affiliations:** ^1^IRCCS Centro Neurolesi Bonino Pulejo, Messina, Italy; ^2^Department of Ophthalmology, IRCCS Ospedale San Raffaele, University Vita-Salute San Raffaele, Milan, Italy; ^3^Department of Clinical and Experimental Medicine, University of Messina, Messina, Italy; ^4^Department of Biomedical Sciences and Morphological and Functional Images, University of Messina, Messina, Italy; ^5^Fresco Institute for Parkinson's & Movement Disorders, NYU-Langone School of Medicine, New York, NY, United States

**Keywords:** diffusion MRI, DTI, CSD, AFD, tractography, white matter quantification, corticospinal tract, arcuate fasciculus

## Abstract

In the last decades, a number of Diffusion Weighted Imaging (DWI) based techniques have been developed to study non-invasively human brain tissues, especially white matter (WM). In this context, Constrained Spherical Deconvolution (CSD) is recognized as being able to accurately characterize water molecules displacement, as they emerge from the observation of MR diffusion weighted (MR-DW) images. CSD is suggested to be applied on MR-DW datasets consisting of b-values around 3,000 s/mm^2^ and at least 45 unique diffusion weighting directions. Below such technical requirements, Diffusion Tensor Imaging (DT) remains the most widely accepted model. Unlike CSD, DTI is unable to resolve complex fiber geometries within the brain, thus affecting related tissues quantification. In addition, thanks to CSD, an index called Apparent Fiber Density (AFD) can be measured to estimate intra-axonal volume fraction within WM. In standard clinical settings, diffusion based acquisitions are well below such technical requirements. Therefore, in this study we wanted to extensively compare CSD and DTI model outcomes on really low demanding MR-DW datasets, i.e., consisting of a single shell (*b*-value = 1,000 s/mm^2^) and only 30 unique diffusion encoding directions. To this end, we performed deterministic and probabilistic tractographic reconstruction of two major WM pathways, namely the Corticospinal Tract and the Arcuate Fasciculus. We estimated and analyzed tensor based features as well as, for the first time, AFD interpretability in our data. By performing multivariate statistics and tract-based ROI analysis, we demonstrate that WM quantification is affected by both the diffusion model and threshold applied to noisy tractographic maps. Consistently with existing literature, we showed that CSD outperforms DTI even in our scenario. Most importantly, for the first time we address the problem of accuracy and interpretation of AFD in a low-demanding DW setup, and show that it is still a biological meaningful measure for the analysis of intra-axonal volume even in clinical settings.

## Introduction

In the last decades diffusion MRI allowed to study non-invasively white matter (WM) by analyzing water molecules diffusion process *in vivo* (1). Diffusion Tensor imaging (DTI) was the first model historically developed to describe tridimensional water anisotropic motion in the brain (2). DTI is based on the estimation of diffusion tensors, i.e., order 3 positive definite matrices: tensor eigensystem describes water apparent diffusivity (the eigenvalues) in three main orthogonal directions (the eigenvectors) (3). Taking into account tensor model, tissues quantification has been characterized over the years by means of several scalar indices, such as fractional anisotropy (FA) and mean diffusivity (MD) (4). These values have been shown to correlate with WM integrity in normal vs. pathological conditions (5, 6).

Although a number of improvements were performed to get more reliable estimates (7–10), tensor model suffers from severe limitations, like the inability to resolve complex fiber configurations within a voxel (11–14). A number of techniques have been thus developed to overcome such limitations, like multi-tensor models (15–17), Q-ball imaging (18), Constrained Spherical Deconvolution (CSD) (19–21) and Diffusion Spectrum Imaging (22).

CSD is a modified high angular resolution diffusion imaging (HARDI) based model (18, 23) that investigates water motion by fitting a set of rotational and spherical harmonics to determine the so-called fiber Orientation Distribution Function (fODF) (21, 22). CSD attempts to individuate all fiber populations insisting over WM voxels based on a representative one-fiber population signal, the so-called response function.

From CSD framework it is possible to derive indices to quantify at voxel-wise level WM fiber bundles (24, 25); compared to DTI based features, these indices are unbiased with respect to the presence of multiple fiber bundles insisting inside the same voxel. In particular, in Raffelt's paper (25) apparent fiber density (AFD) has been introduced: based on the properties of diffusion signal, AFD attempts to estimate intra-axonal volume fraction (26) of each distinct fiber bundle insisting over WM voxels. AFD has been recently successfully applied in normal as well as pathological conditions (27, 28).

In current literature, like other HARDI based methods, CSD and AFD are recommended to be employed in diffusion datasets with relatively high *b*-values (3,000 s/mm^2^) and a minimum of 45 distinct encoding diffusion directions (19). At the same time, other tissue quantifications techniques, e.g., NODDI (29) or CHARMED (30), involve schemes with multiple shells (i.e., multiple *b*-values).

Currently, especially in clinical environments, a huge number of acquisition protocols make use of datasets with lower *b*-values (1,000–1,200 s/mm^2^), and fewer unique directions (~30). This happens because of the old machine used, or mainly because they require less time to be accomplished. Time factor is indeed of huge importance when dealing with patients, who are usually less tolerant and collaborative due to their particular conditions. In such scenarios, the common approach is to stick with tensor model and related analyses. Despite that, in the last years an emerging number of studies applied CSD on datasets derived from a setup that is below the recommended standards, see for instance (31–33).

In this study we aim at systematically investigating the amount of information achievable within the CSD framework on real data with a very low-demanding setting, i.e., when employing a single shell (*b*-value = 1,000 s/mm^2^) and 30 unique diffusion gradient directions. To this end, we compared CSD outcomes with DTI model ones, which is the gold standard with those settings. We performed tractographic reconstruction and related tract-based ROI analysis (TB-RA) of two well-known pathways, namely the Corticospinal Tract (CST) and the Arcuate Fasciculus (AF). Deterministic as well as probabilistic tractography were implemented and compared based both on DTI and CSD models (21, 34–37). By means of multivariate statistics we subsequently analyzed tissue quantification dependence on the diffusion model adopted as well as on the threshold levels applied to noisy tractographic maps. Most importantly, for the first time with the abovementioned hardware settings, we focus on the biological meaningfulness of AFD to understand whether it can be still considered a valid surrogate of intra-axonal volume fraction. Eventually, based on the TB-RA, we investigate the nature of the relationship between AFD and FA.

## Materials and methods

### MRI protocol and pre-processing

Thirteen right-handed healthy subjects (6 women and 7 males, mean age 32.4 years, age range 25–42 years) without any history of neurological disease were recruited for this study. Before MRI acquisitions, each participant signed a written informed consent. The entire study was approved by the Ethical Committee of IRCCS Centro Studi Neurolesi “Bonino-Pulejo”; investigation has been conducted in accordance with the Declaration of Helsinki. MRI acquisition protocol was performed on a 3T Achieva Philips scanner (Philips healthcare, Best, The Netherlands) mounting a 32-channel SENSE head coil. Following datasets were collected:

- Structural T1-weighted 3D high-resolution Fast Field Echo (FFE) sequence; TR = 25 ms; TE = 4.6 ms; flip angle 30°; FOV 240 × 240 mm^2^; in plane reconstruction matrix 240 × 240; voxel size 1 × 1 × 1 mm^3^.
- Single-shot echo-planar diffusion weighted sequence (DW-EPI); TR = 11,884 ms; TE = 54 ms; FOV 240 × 240 mm^2^; scan matrix 120 × 120; in-plane resolution 2 × 2 mm^2^, axial slice thickness 2 mm without inter-slice gap. One unweighted b0 volume and 30 diffusion encoding directions covering a half sphere were acquired. *B*-value was set to 1,000 s/mm^2^.

For each subject, motion and eddy current distortions artifacts occurring on DW volumes were corrected by means of *eddy* FSL tool (http://fsl.fmrib.ox.ac.uk/fsl/fslwiki/). Rotational part of transformations originating from correction process was subsequently applied to gradient directions. To obtain an estimation of AFD that could be comparable across subjects, the same preprocessing pipeline suggested in Raffelt et al. (25) was adopted. Therefore, b0 volume was used to estimate a multiplicative bias field by means of *fast* FSL routine. After applying bias field to b0 and DW images, all volumes were normalized by dividing by the median b0 intensity measured on an WM mask. T1w image was later on co-registered to preprocessed DWs following a scheme previously reported (38): in brief, CSF probability maps were estimated separately for b0 and T1 images by means of *New Segment* option of SPM8 (https://www.fil.ion.ucl.ac.uk/spm/). B0-based CSF probability map was then up-sampled to the same resolution of T1; *flirt* and *fnirt* FSL commands were subsequently employed to warp T1-based CSF probability map to the b0 based one. Estimated warping field was eventually applied to structural scan.

### Tractography

Tensor estimation and DT based tractography were accomplished using CAMINO package (39) (http://cmic.cs.ucl.ac.uk/camino/). In this context, tensors fitting was computed by means of a non-linear constrained procedure (7, 8). After terminating DT fitting procedures, WM voxels were inspected in order to detect possible unreal eigenvalues; any implausible eigenvalue was found in the datasets.

CSD based computations were performed by means of MRtrix software package (40), version 3 (www.mrtrix.org). Subject-specific response functions were firstly estimated for each DW dataset (41), and then averaged to produce a study-specific averaged response function. This latter was subsequently used to estimate fODF in each diffusion dataset; for those computations, maximal harmonic order was set to 6.

Deterministic and probabilistic tractographic reconstructions were performed using *track* CAMINO command and *tckgen* MRtrix command for DTI and CSD, respectively.

For AF tracking, a single seed ROI (s-ROI) was placed following a procedure previously suggested (42, 43). For CST tracking, medial (med-CST) and lateral (lat-CST) CST portions were separately reconstructed. To this end, two cortical s-ROIs related to the pathways of interest were manually defined by an expert radiologist (M.G.). When seeding for either the medial or lateral portion of CST, the other s-ROI was included as Region of Avoidance (ROA); in addition, another ROA was placed to impede reaching of the contralateral hemisphere. All tractographic reconstructions were allowed to propagate within a mask comprising WM voxels: for each subject, this mask was created based on segmentation of coregistered T1 as provided by *New Segment* SPM8 tool. Prior running tractography, WM mask was moderately dilated (npass = 3 option of *maskfilter* MRtrix command) to permit to the streamlines to reach GM.

For deterministic DTI and CSD tracking, a single seed was initialized for each voxel of related s-ROI, whereas 100 streamlines were generated from each voxel being part of seed ROI for probabilistic tracking.

For deterministic DTI (d-DTI), directional clues were provided by eigenvector coupled to highest eigenvalue using FACT algorithm (44). For probabilistic DTI tracking (p-DTI), uncertainty in principal diffusion direction (PDD) was determined using the probabilistic index of connectivity (PICo) (35); Bingham distribution was used to estimate PDDs distribution. For both reconstruction methods, Runge–Kutta 4th order for streamline direction interpolation step was used, as well as an overall FA threshold of 0.2 together with an angular threshold of 60° to avoid unrealistic trajectories for streamlines.

For deterministic CSD (d-CSD), direction corresponding to highest fODF peak was used; for probabilistic CSD (p-CSD) tractography we used an algorithm described in Jeurissen et al. (37). Ifod2 interpolation scheme (45) was employed to interpolated fODF peak directions at each reconstruction step, whose length was set to 0.2 mm. Minimal fODF amplitude and angular threshold were set to 0.15 and 60°, respectively.

### Analysis of tractographic maps and DTI parameters

As first part of our experiment, we aimed at measuring performances of different tractographic methods in reconstructing WM fiber bundles under examination. To this end, we estimated Overlap Fraction (OF) (46, 47). OF was measured via the following formula:

$$\begin{array}{l} OF=\frac{100^{*}V_{target}\cap V_{reference}}{\left(V_{reference}\right)}\% \end{array}$$

in which *V*_*reference*_ represents a tract-based volume defined on the basis of p-CSD tractographic outcome, whereas *V*_*target*_ is the tract-based volume estimated for all the other methods (d-DT, p-DTI, and d-CSD). Those tract-based volumes were created by generating corresponding track density images (TDIs) (48). TDI is a map in which each voxel is assigned a value corresponding to the number of streamlines passing through it. To address the issue of false positive artifacts in streamlines output, OFs calculation were repeated after thresholding TDIs at different density levels. For each reconstructed pathway and method used, maximal density was calculated; then, only voxels whose density was above a given percentage of that maximal value were retained. Since streamlines trajectories are known to be prone to false positive artifacts, we thresholded TDIs at the following percentages: 0% (raw TDIs), 1, 5, 10% of the maximal density for that reconstruction. OF analyses were performed in normalized MNI space: for each subject, FA maps were warped to match *FMRIB58_FA* template by using *flirt* and *fnirt* commands. Estimated warping fields were later on applied to TDIs to obtain those maps in normalized space.

Tensor features were computed by means of in-house algorithms written with MATLAB software package (www.mathworks.com/products/matlab/), release 2015. Thresholded TDIs were used as masks from which gathering and averaging following measures: FA, MD, Linear Coefficient (CL), Planar Coefficient (CP), Spherical Coefficient (CS), Axial (AD), and Radial (RD) diffusivity. Tensor based maps were created in native spaces and later on warped using the same warping field previously estimated to project TDIs into the MNI space.

Impact of tractographic techniques and cutoff levels on tensor features was investigated by means of multivariate Wilks' Lambda tests using *cutoff* (levels: 0, 1, 5, 10%) and *method* (levels: d-DTI, p-DTI, d-CSD, p-CSD) as within-subjects factors; all analyses were conducted using SPSS statistical package (http://www-01.ibm.com/software/), release 22. Where necessary, Bonferroni correction was applied in *post-hoc* analyses to correct for multiple comparisons in order to get a global significance type-I error of 0.05.

### Estimation of intra-axonal volume fraction and comparison with FA

In the second part of our experiment, we estimated and analyzed intra-axonal volume fractions in our datasets. This task has been accomplished in four steps: (i) voxels having a relatively high FA were isolated; (ii) for each voxel, the highest fODF lobe amplitude (the peak) and the corresponding peak direction were detected. (iii) Assuming that fiber population is pointing along direction provided by highest fODF peak, intensity of DW volume whose gradient direction was the more perpendicular to highest peak direction was extracted for each voxel. This intensity should correspond to DW signal radial to the fiber population. (iv) Eventually, intensity was normalized by the intensity of the b0 signal acquired in the same voxel. Consistently with (25), such normalized value has been interpreted as an estimate of intra-axonal volume fraction for that given voxel. Steps i-iv were repeated for four different FA cutoff levels: 0.7, 0.75, 0.8, 0.85. Selection of voxels based on FA level, fODF estimation and peak detection were done by using MRtrix. Final data were gathered and visualized by means of Matlab.

A deeper investigation of AFD has been accomplished by performing comparison with one of the mostly known DTI parameters, namely FA. At this stage the focus was placed on p-CSD based reconstructions. Of note, we worked in the native space of our subjects to avoid biases in normalization process of fODF lobes (28). Firstly, AFD was calculated in all voxels from which at least one streamline was passing through. To this end *afdconnectivity* MRtrix command was adopted. Subsequently, for each subject and WM pathway analyzed, both linear and cubic polynomial fits were applied to see how FA was related to AFD. Fitting results were later on compared each other by estimating the following F-statistic:

$$\begin{array}{l} F=\frac{\left(SS_{smal}-SS_{big}\right)/\left(df_{small}-df_{big}\right)}{SS_{big}/df_{big}} \end{array}$$

where *SS*_*smal*_ and *SS*_*big*_ are the residual sum of squares of linear and cubic models having *df*_*smal*_ and *df*_*big*_ degrees of freedom, respectively. With the goal to individuate the most plausible pattern between the two measures, the value above described was observed on an F distribution with *df*_*big*_−*df*_*small*_ and *df*_*big*_ degrees of freedom, respectively. A linear fit would of course indicate a linear relationship; if a cubic “S-shape” fit would instead better represent FA vs. ADF profiles, a non-linear relationship should be inferred between AFD and FA, in particular when analyzing lowest and highest FA values. Curve fitting (robust estimation) and F-tests were carried out by means of tools available within Matlab.

The pipelines of both experiments are shown for visualization purposes at the end of the manuscript (see Figure 5).

## Results

### Tractographic reconstruction accuracy and robustness

**Figure 1:**
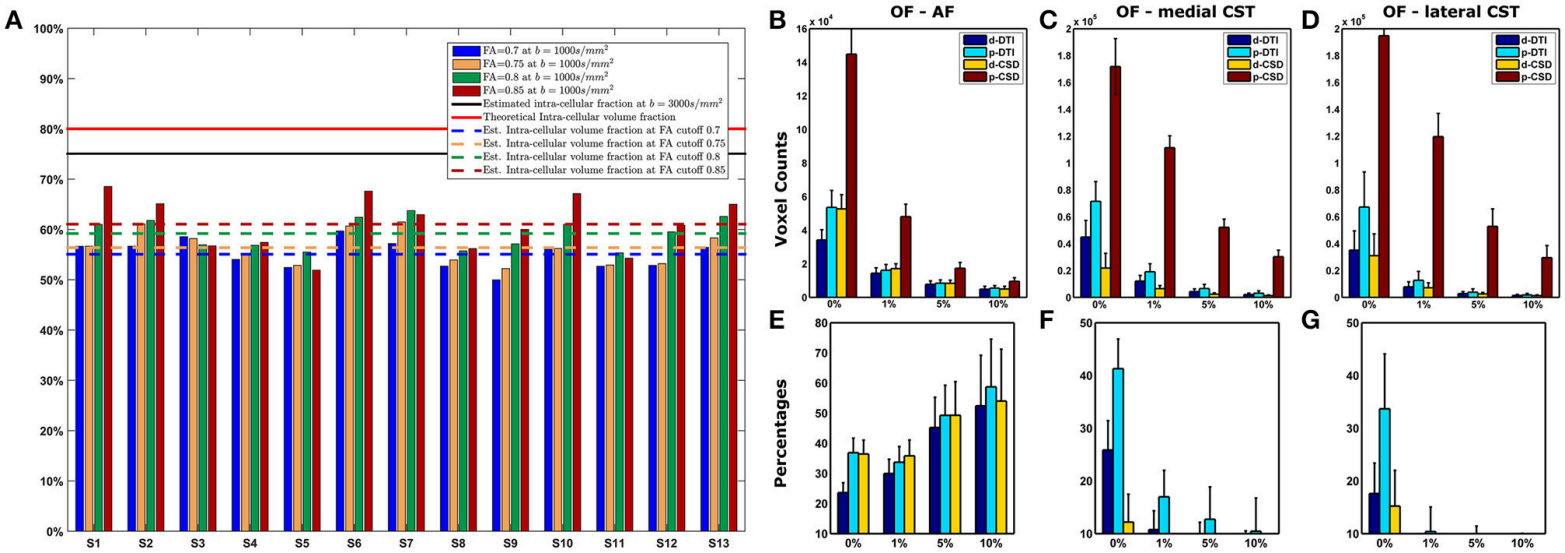
Intra-axonal volume fraction and OF analysis. **(A)** Intra-axonal volume fraction estimated with a *b*-value of 1,000 s/mm^2^. Theoretic limit and average estimates obtained on datasets with *b*-values of 3,000 s/mm^2^ are shown (continuous lines); mean estimates averaged over all subjects in this study are shown with dashed lines, one for each FA cutoff. **(B–E)** Overlap Fraction analysis. Voxels count for each method and density cutoff is shown for AF **(B)**, medial CST **(C)**, and lateral CST **(D)** portion reconstructions; data reported are averaged over subjects and between left and right hemispheres. At the same time, OF analysis (**E–G**, based on probabilistic CSD reconstruction used as reference (brown bar), shows how both deterministic (blue) and probabilistic (cyan) DTI cause notable underestimation either for AF **(E)**, medial **(F)**, and lateral **(G)** CST portions. Whiskers represent one standard deviation. OF, Overlapping fraction; AF, Arcuate Fasciculus; CST, Corticospinal Tract.

Results of OF analysis are reported in Table 1 and shown in Figures 1B–G for visualization purposes: in Table 1, OFs were averaged over subjects and between hemispheres. For all WM fasciculi investigated, probabilistic CSD tractography determined the densest and widest reconstructions in all subjects. As expected, raw count of voxels traversed by streamlines decreased as density cutoff increased (Figures 1B–D).

**Table 1:** OF analysis results.

**Fasciculus**	**Method**	**Cutoff level**							
		**0%**		**1%**		**5%**		**10%**	
		**Mean**	**Std**	**Mean**	**Std**	**Mean**	**Std**	**Mean**	**Std**
AF	d-DTI	23.61	3.28	29.94	4.76	45.21	10.03	52.40	16.78
	p-DTI	36.87	4.80	33.71	5.19	49.25	9.96	58.71	15.84
	d-CSD	36.44	4.61	35.82	5.25	49.27	11.17	54.00	17.20
Medial CST	d-DTI	25.86	5.56	10.75	3.58	8.26	3.87	7.00	3.54
	p-DTI	41.28	5.63	16.98	5.02	12.72	6.13	10.45	6.29
	d-CSD	12.18	5.29	5.77	1.94	4.59	1.68	4.19	1.63
Lateral CST	d-DTI	17.60	5.77	6.38	2.55	5.23	2.09	5.02	1.88
	p-DTI	33.67	10.40	10.39	4.67	7.32	4.11	6.45	3.59
	d-CSD	15.22	6.76	5.84	2.56	4.80	1.86	4.60	1.84

*For each density cutoff levels and pathway investigated. Overlap Fraction measures the amount of tract preserved with respect to reference p-CSD tractographic reconstructions*.

When investigating AF results, differences between probabilistic CSD based reconstruction and other methods tended to decrease as density increased; however, all OFs resulted below 60% (Figure 1E). A different behavior was instead observed for medial and lateral CST reconstructions if compared to AF results (Figures 1F,G): in those situations, differences between p-CSD and OFs measured with other techniques tended to slightly increase as density cutoff increased. Analysis of lateral and medial CST portions determined similar results.

**Figure 2:**
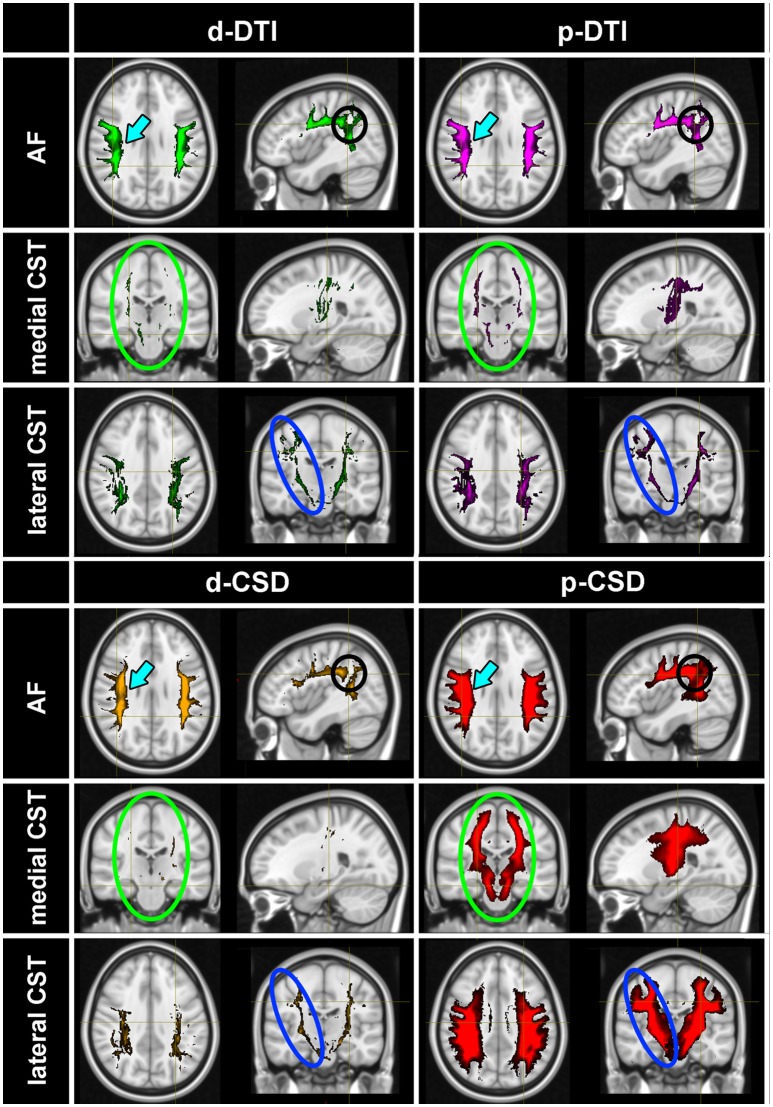
Tractographic results. TDIs maps (thresholded at 5% of the maximal density) obtained from all subjects were warped and averaged in the MNI space for each method: p-CSD (red) related TDIs show the best results for AF, either in terms of tract definition (cyan arrows) as well as in the depiction of its curvature (black circles). p-CSD clearly outperforms DTI, shown with green and purple maps for deterministic and probabilistic results, in reconstructing medial (green circles) and lateral (blue circles) CST portions. Deterministic CSD (orange) is inadequate as well to produce robust reconstructions. AF, Arcuate Fasciculus; CST, Corticospinal Tract.

Tractographic results obtained by thresholding at 5% of maximal density are shown for visualization purposes in Figure 2. TDIs maps obtained from all subjects were warped and averaged into the MNI space and overlaid onto a reference T1w template; maps were intensity scaled to maximize visibility. p-CSD related TDI (in red) provided the best result for AF, either in terms of tract definition (cyan arrows) as well as in the depiction of its curvature (black circles appearing in Sagittal views of Figure 2). Huge differences between p-CSD with respect to d-DTI (green maps), p-DTI (purple maps) related density images were observed when comparing reconstruction of medial (green ellipses) as well as lateral (blue ellipses) CST portions. All these maps showed how CSTs were well described by p-CSD, whereas poor results were obtained by the other methods. Deterministic CSD (orange map) resulted inadequate for providing robust reconstructions.

### TB-RA

**Figure 3:**
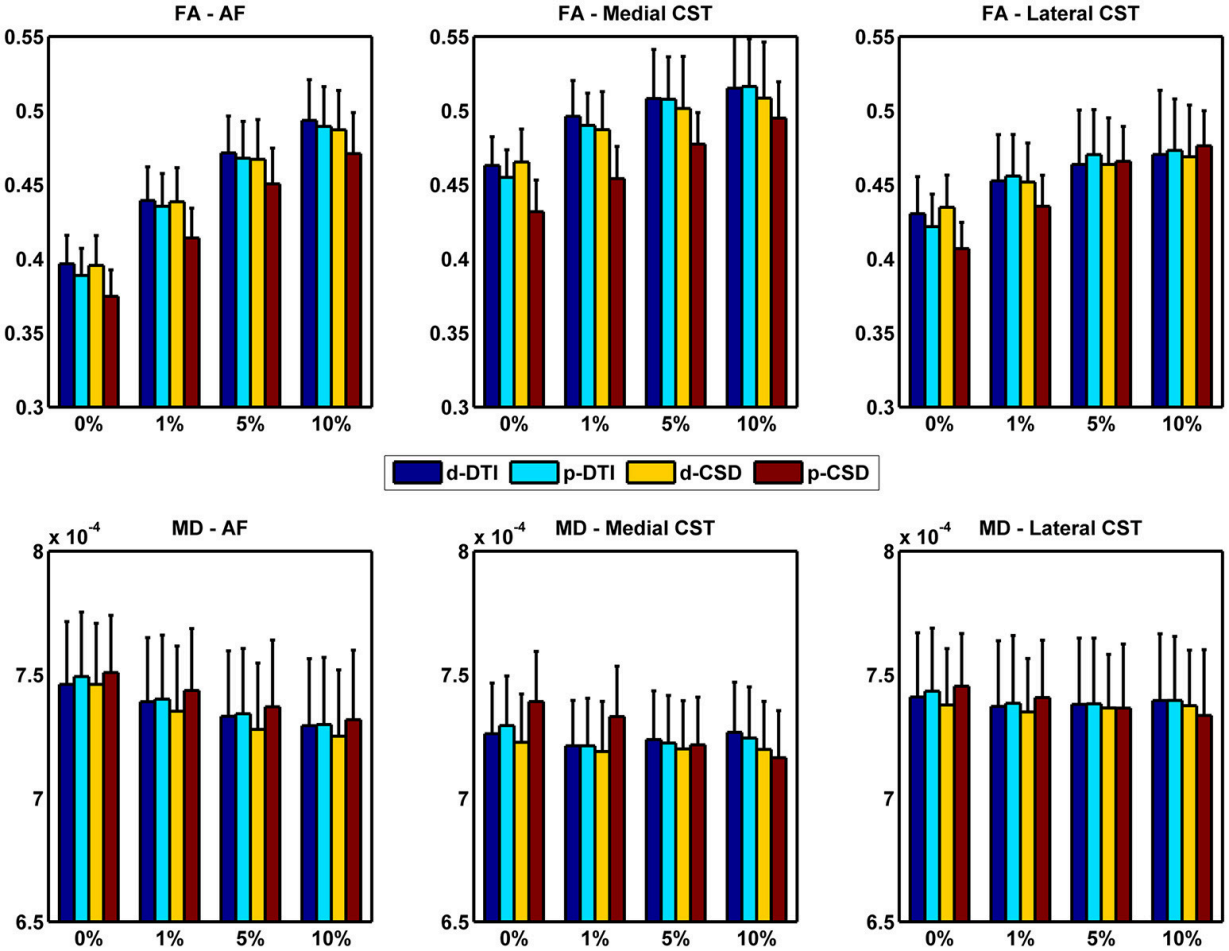
FA and MD variations due to cutoff used and reconstruction technique adopted. Whiskers represent one standard deviation. AF, Arcuate Fasciculus; CST, Corticospinal Tract.

In this section we investigated impact of tractographic algorithms on TB-RAs. In Figure 3 we show FA and MD variation for AF, medial and lateral CST portions for all density cutoff levels and reconstruction algorithms. Details of multivariate statistical analyses results are reported in Table 2. The strong effects provided both by *cutoff* and *method* factors on the estimation of average FA could be clearly observed: for all WM bundles under examination, *cutoff* factor significantly influenced FA outcome. *Post-hoc* analyses confirmed a positive linear trend either for AF (*F* = 652.755, uncorrected *p* = 7.87E-12), medial CST (*F* = 122.492, uncorrected *p* = 1.18E-07), and lateral CST (*F* = 174.799, uncorrected *p* = 1.63E-08) portions. At the same time, *post-hoc* analyses on *method* sub-levels showed a significant decrease of p-CSD based FA with respect to all other techniques, with the only exception of lateral CST based results for which no significant differences were observed after Bonferroni correction.

**Table 2:** Tractograms cutoff and tractographic algorithm impact on Diffusion Tensors features.

**Parameter**	**WM bundle**	**Multivariate Test: Wilks' Lambda**					
		**Effect**	**Value**	***F***	**Hypothesis df**	**Error df**	**Uncorrected *p*-value**
FA	AF	*Cutoff*	0.013	248.212	3.000	10.000	1.10*E*−09^**^
		*Method*	0.048	66.103	3.000	10.000	6.76*E*−07^**^
	Medial CST	*Cutoff*	0.075	41.149	3.000	10.000	6.19*E*−06^**^
		*Method*	0.170	16.317	3.000	10.000	3.52*E*−04^**^
	Lateral CST	*Cutoff*	0.035	92.692	3.000	10.000	1.34*E*−07^**^
		*Method*	0.439	4.261	3.000	10.000	0.035*
MD	AF	*Cutoff*	0.067	46.772	3.000	10.000	3.43*E*−06^**^
		*Method*	0.058	54.244	3.000	10.000	1.72*E*−06^**^
	Medial CST	*Cutoff*	0.117	25.113	3.000	10.000	5.68*E*−05^**^
		*Method*	0.299	7.800	3.000	10.000	0.006^**^
	Lateral CST	*Cutoff*	0.215	12.156	3.000	10.000	0.001^**^
		*Method*	0.637	1.897	3.000	10.000	0.194
CL	AF	*Cutoff*	0.022	149.498	3.000	10.000	1.32*E*−08^**^
		*Method*	0.082	37.441	3.000	10.000	9.54*E*−06^**^
	Medial CST	*Cutoff*	0.087	34.955	3.000	10.000	1.30*E*−05^**^
		*Method*	0.128	22.714	3.000	10.000	8.78*E*−05^**^
	Lateral CST	*Cutoff*	0.040	80.661	3.000	10.000	2.62*E*−07^**^
		*Method*	0.232	11.016	3.000	10.000	0.002^**^
CP	AF	*Cutoff*	0.067	46.360	3.000	10.000	3.6*E*−06^**^
		*Method*	0.270	9.007	3.000	10.000	0.003^**^
	Medial CST	*Cutoff*	0.027	118.951	3.000	10.000	4.03*E*−08^**^
		*Method*	0.988	0.039	3.000	10.000	0.989
	Lateral CST	*Cutoff*	0.071	43.875	3.000	10.000	4.61*E*−06^**^
		*Method*	0.245	10.294	3.000	10.000	0.002^**^
CS	AF	*Cutoff*	0.013	257.857	3.000	10.000	9.12*E*−10^**^
		*Method*	0.068	45.853	3.000	10.000	3.76*E*−06^**^
	Medial CST	*Cutoff*	0.076	40.320	3.000	10.000	6.80*E*−06^**^
		*Method*	0.228	11.263	3.000	10.000	0.002^**^
	Lateral CST	*Cutoff*	0.040	79.778	3.000	10.000	2.76*E*−07^**^
		*Method*	0.839	0.638	3.000	10.000	0.607
AD	AF	*Cutoff*	0.052	61.140	3.000	10.000	9.78*E*−07^**^
		*Method*	0.069	44.727	3.000	10.000	4.22*E*−06^**^
	Medial CST	*Cutoff*	0.162	17.222	3.000	10.000	2.82*E*−04^**^
		*Method*	0.202	13.207	3.000	10.000	8.21*E*−04^**^
	Lateral CST	*Cutoff*	0.052	60.935	3.000	10.000	9.94*E*−07^**^
		*Method*	0.520	3.071	3.000	10.000	0.078
RD	AF	*Cutoff*	0.019	175.575	3.000	10.000	6.03*E*−09^**^
		*Method*	0.060	52.496	3.000	10.000	2.00*E*−06^**^
	Medial CST	*Cutoff*	0.098	30.574	3.000	10.000	2.38*E*−05^**^
		*Method*	0.158	17.784	3.000	10.000	2.47*E*−04^**^
	Lateral CST	*Cutoff*	0.049	64.133	3.000	10.000	7.80*E*−07^**^
		*Method*	0.337	6.564	3.000	10.000	0.010*

*Dependence of DT parameters on cutoff and reconstruction methods (Wilks' Lambda tests). Asterisks indicate significance at 0.05 (one asterisk) and 0.01 (two asterisks) levels*.

A similar trend was found when investigating MD variation. Like for FA, *cutoff* factor resulted always significant, and *post-hoc* analyses confirmed a significant negative trend either for AF (*F* = 124.023, uncorrected *p* = 1.11E-07), medial CST (*F* = 30.866, uncorrected *p* = 1.25E-04), and lateral CST (*F* = 23.556, uncorrected *p* = 3.96E-04) portions. *Method* factor resulted always significant, except when inspecting reconstructions of lateral CST. In a symmetric fashion with respect to FA, *post-hoc* analyses showed that MD averaged from voxels traversed by p-CSD led to systematically higher values if compared to other techniques.

Analyses of Westin indices as well as of AD and RD yielded similar results (see Table 2).

### AFD estimation results and comparison with FA

Results of estimated intra-axonal volume fraction in our datasets were shown in Figure 1A. Estimated fraction was below both the theoretical intra-cellular volume fraction (26) (80%) as well as the intra-axonal volume fraction reported in Raffelt et al. (25), where an average value of 75% was found based on *b* = 3,000 s/mm^2^. In our data, we observed a clear volume fraction dependence on FA cutoff level for voxels selection: indeed, based on FA cutoff of 0.7 we estimated an average volume fraction of 55.16% (*SD* = 2.90%); for 0.75 FA cutoff, average volume fraction was 56.45% (*SD* = 3.34%). For voxels having FA ≥ 0.8, the estimated intra-axonal volume fraction was 58.97% (*SD* = 3.00%). Eventually, we estimated an average intra-axonal volume fraction of 60.51% (*SD* = 4.95%) based on voxels having FA equal or above 0.85. These results are shown in Table 3 together with the percentages of voxels involved in the calculation.

**Table 3:** Intra-axonal volume fraction.

	**FA Cutoff Level**			
	**0.7**	**0.75**	**0.8**	**0.85**
Estimated intra-axonal volume fraction	55.16% (2.90%)	56.45% (3.34%)	58.97% (3.00%)	6.51% (4.95%)
Voxels percentage involved in calculation, compared to a whole brain mask	3.69% (0.72%)	2.53% (0.50%)	1.70% (0.33%)	1.13% (0.23%)

*For each FA cutoff level (0.7, 0.75, 0.8, 0.85), average intra-axonal volume fraction has been estimated from all subjects; standard deviations are reported between brackets. In the bottom part, percentage of voxels involved in calculations at each cutoff level is reported; such percentage is related to foreground voxels of a whole brain mask obtained on b0 volumes. Even in this case, standard deviation is reported between brackets*.

**Figure 4:**
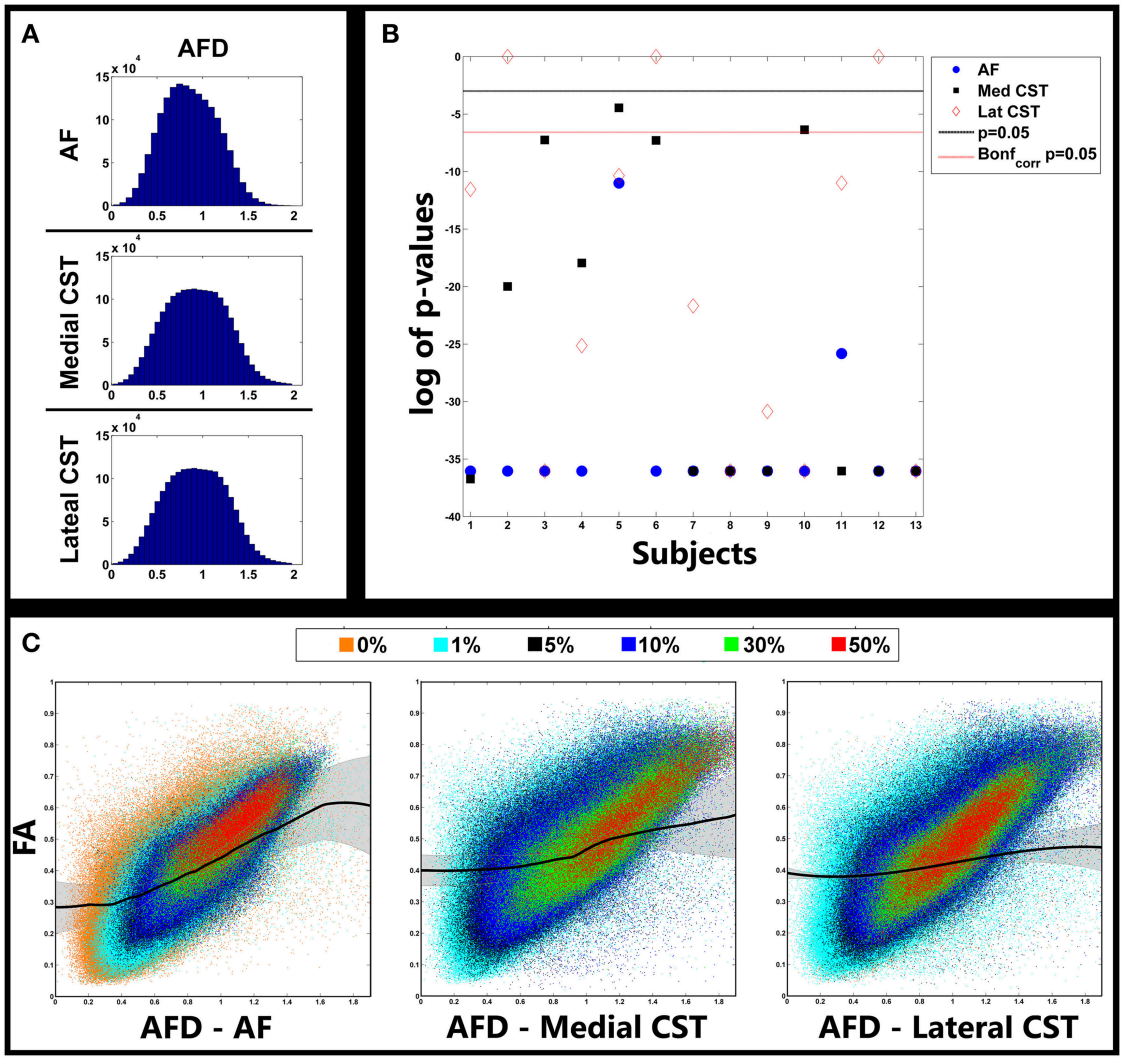
Relationship between AFD and FA. **(A)** Distribution of AFD gathered from fiber pathways investigated in all subjects. **(B)** (uncorrected) *p*-values of model selection tests between cubic and linear fits of FA vs. AFD curves for each subject and pathway. Lines represent type I error level of 0.05 both uncorrected (black) and corrected (red dotted) for multiple comparisons via Bonferroni correction. Significant values indicate that cubic fit outperformed linear one. **(C)** Scatterplots showing relationship between AFD and FA. Colors represent different cutoff levels. Black lines represent cubic fit averaged over all subjects. Shaded lines represent standard deviations. AFD, Apparent Fiber Density; AF, Arcuate Fasciculus; CST, Corticospinal Tract.

The relationship between AFD and FA in the investigated pathways are shown in Figure 4. In Figure 4A we reported histograms of AFDs gathered from all subjects separately for AF, medial and lateral CST portions. Despite the lack of a nominal limit, AFD values ranged primarily between 0 and 2. Similar distributions were observed for all pathways, with a moderate smaller mode for AF (around 0.7); modes for medial and lateral CST based AFDs were instead around 0.9.

**Figure 5:**
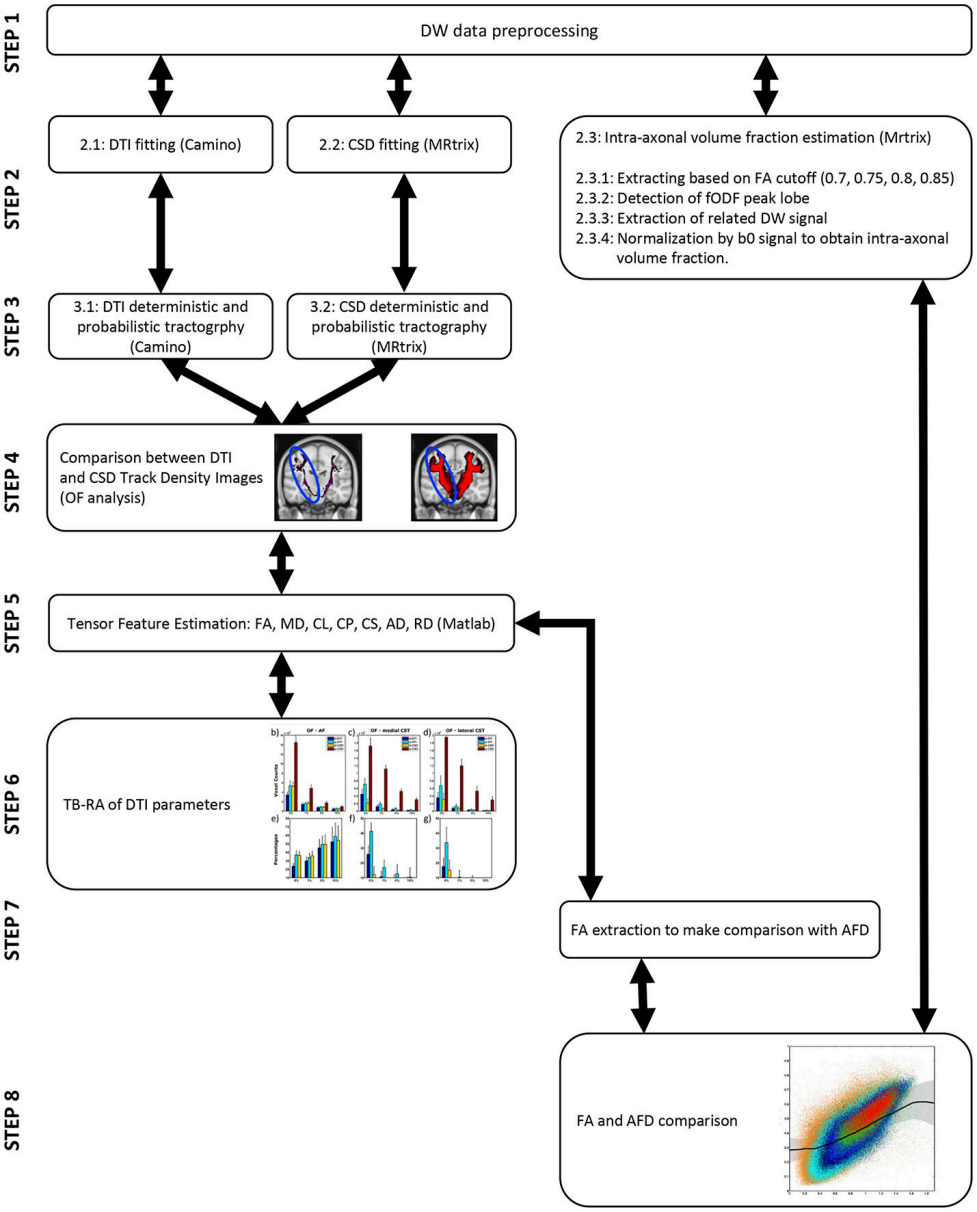
Pipeline of all the experiments conducted. On the left steps leading to tractographic reconstruction comparisons and TB-RA (steps 2–6) are reported. On the right steps leading to AFD analysis (steps 2, 7, 8) are instead shown.

In Figure 4B statistical results of model selection tests were shown for all subjects and pathways; to help visualization, *p*-values were reported on a log scale. For most of the comparisons, the polynomial cubic fit was the one that better represented the data, thus underlying the non-linear nature of the relationship. A few exceptions were observed in three subjects when reconstruction the lateral portion of CST, and in one subject (S5) in which the linear fit over-performed the cubic one. All *p*-values are reported in Table 4.

**Table 4:** Relationship between AFD and FA.

**Subject**	**AF**	**M-CST**	**L-CST**
	**Uncorrected*p*-values**	**Uncorrected *p*-values**	**Uncorrected *p*-values**
S1	< 0.001^*^	< 0.001^*^	< 0.001^*^
S2	< 0.001^*^	< 0.001^*^	1.000
S3	< 0.001^*^	0.001^*^	< 0.001^*^
S4	< 0.001^*^	< 0.001^*^	< 0.001^*^
S5	< 0.001^*^	0.012	< 0.001^*^
S6	< 0.001^*^	0.001^*^	1.000
S7	< 0.001^*^	< 0.001^*^	< 0.001^*^
S8	< 0.001^*^	< 0.001^*^	< 0.001^*^
S9	< 0.001^*^	< 0.001^*^	< 0.001^*^
S10	< 0.001^*^	0.002^*^	< 0.001^*^
S11	< 0.001^*^	< 0.001^*^	< 0.001^*^
S12	< 0.001^*^	< 0.001^*^	1.000
S13	< 0.001^*^	< 0.001^*^	< 0.001^*^

*Uncorrected p-values coming out of F-tests comparing cubic over linear fit. Asterisks indicate that cubic model fit was the best choice with a p-value lower than 0.05 type-I threshold after Bonferroni correction. AF, Arcuate Fasciculus; M-CST, Medial Corticospinal Tract; L-CST, Lateral Corticospinal Tract*.

A better understanding of complex covariance between AFD and FA can be appreciated in Figure 4C. In each panel, scatters represented FA vs. AFD values gathered from all subjects for each given pathway. Colors highlight voxels belonging to different TDIs cutoff levels: 0% (orange), 1% (cyan), 5% (black), 10% (blue), 30% (light green), and 50% (red).

First of all, as expected, as long as cutoff increases FA tends to increase and its variance to decrease. The lower prevalence of orange clouds for AF over the panels reporting data collected from medial and lateral CST portions is likely due to the substantial overlap of 0 and 1% clouds in the latter two situations. This latter is a sign of the higher variability when reconstructing AF.

Overall, a clear positive trend was observed; importantly, in the bottom right part of all the panels, we could see a huge number of points for which FA was relatively low (between 0.1 and 0.4), whereas AFD substantially increased. This secondary trend corresponded to voxels over which crossing or branching multiple fibers insisted.

Black colored curves showed results of the cubic fit averaged over all subjects (Figure 4). Shadow represents standard deviation. It is clear how at the borders a strictly linear relationship between FA and AFD did not hold. This phenomenon was especially evident for AF and medial portion of CST, and confirmed that AFD was able to span a higher range of fiber geometries configurations; such geometries could not in turn be exhaustively represented by FA because of its known limitations. Since for three subjects a linear fit betted represented the data, by averaging data obtained from lateral CST, a more flat curve was estimated (Figure 4).

## Discussion

In this study we compared CSD and DTI model ability in reconstructing and quantifying two well-known fiber pathways, the Corticospinal Tract and the Arcuate Fasciculus.

While other pathways could have been investigated, we preferred to focus on CST and AF for two reasons. First of all their anatomical configurations, i.e. their width as well as the smooth changes of direction along their courses, should in principle ease reconstructions provided by the simpler DTI model, thus rendering a comparison with CSD consistent and robust. Secondly, those pathways have been historically widely investigated by means of DTI model to probe motor and associative brain networks, both in healthy and pathological conditions.

### CSD and DTI models

It is known that DTI is unable to resolve multiple fibers configurations such as bending, kissing, and crossing fibers (3, 5); more complex models are therefore needed (18, 19, 22). Within the CSD framework, the estimation of the fODF (19, 21) allows to properly model diffusion signal; successful applications were obtained in healthy cohorts as well as in pathological populations (27, 30, 48).

AFD (25) is a parameter developed with the aim to provide an estimate of intra-axonal volume fraction; it was shown to be in close correspondence with intra-axonal volume fraction as measured in Syková and Nicholson (26).

CSD framework, along with AFD, is recommended to be applied by using a *b*-value of at least 3,000 s/mm^2^ and a minimum of 45 unique diffusion encoding gradient directions (19). Other tissue quantification parameters, like those coming from CHARMED (30) or NODDI (29), require multi-shell acquisitions as well.

From the other side, DTI is traditionally employed with lower *b*-values (1,000–1,200 s/mm^2^) and lower number of distinct diffusion directions (30–35). In the last years, CSD was however extensively applied on datasets with hardware constraints closer to those adopted for DTI studies (22, 49–51).

In this study we investigated CSD based performances in comparison to the standard DTI model when very minimal technical requirements are used (*b*-value = 1,000 s/mm^2^, 30 unique diffusion encoding directions). In this scenario, particular importance was given to the applicability and biological meaningfulness of AFD.

### Tractographic reconstruction and tensor features analysis

OF analysis confirmed that, if compared to other approaches tested here, probabilistic CSD based tractography determined the densest and widest reconstructions in all subjects investigated.

Differences between p-CSD and other techniques (d-CSD, d-DTI, and p-DTI) slightly decreased as density increased when analyzing AF reconstructions in all subjects (Figure 1).

Furthermore, when investigating tractographic reconstruction of CST, we observed that DTI model markedly underestimated CST if compared to p-CSD (Figure 1). Unlike for AF, differences between p-CSD based TDIs and OFs measured with other techniques tended to increase as density cutoff increases. This is a sign of the fact that p-CSD depicts CST in a more homogeneous way if compared to other techniques, in which a denser kernel pathway is likely reconstructed. It is worth to remark that the reconstruction of lateral and medial CST portions led to similar results.

The improvement achieved by probabilistic CSD can be visually appreciated in Figure 2, in which the warped TDI maps coming from all subjects were averaged and over-imposed onto a T1w template in MNI space.

Altogether those results are consistent with previous literature (13, 52, 53), and confirm that probabilistic CSD is a useful instrument for a more consistent tractographic reconstruction in contexts like pre-surgical planning (13, 54), in case-control studies or in longitudinal analyses.

We furthermore demonstrated that both the tractographic *method* adopted and the *cutoff* chosen to threshold TDIs strongly influence reconstructions outcomes used to perform TB-RA of diffusion tensor indices. This statement was confirmed by multivariate statistical analysis (Table 2).

Regardless to the pathway reconstructed, FA smoothly increased as density cutoff increased, whereas MD decreased. These results are not unexpected: an increasing cutoff is indeed likely going to preserve voxels containing a major dominant fiber population insists, thus explaining both FA and MD behaviors (24).

*Post-hoc* analyses showed that FA was lower when estimated from voxels detected via p-CSD; this situation was expected, since p-CSD involves voxels in which multiple fiber populations insists over, subsequently causing a decrease of FA average. Symmetrically to what it was observed for FA, *post-hoc* analyses showed that MD averaged from voxels traversed by p-CSD was significantly higher when compared to MD estimated on the basis of all other techniques. Variation of Westin indices as well as AD and RD led to similar conclusions.

As already abovementioned, p-CSD involves in TB-RA a higher number of voxels with multiple fiber bundles insisting over them. As we have shown, this situation impacts tensor indices estimation, and therefore might induce to carefully consider p-CSD applicability in pathological conditions where well-established variations (e.g., FA decrease) are traditionally considered linked to WM damages. A possible scenario is the following: if two distinct fiber populations were to insist over the same voxel, and only one of them were damaged by a given disease, we might detect overall FA increase in the patient if compared to a control. In TB-RA context, by summing over all possible voxels, the likelihood of not detecting differences in case-control studies might lead to erroneous results. One might think that the use of tensor based tractography may prevent such circumstances to happen. However, this is not the case because the same situation would apply even if DTI model were adopted: it is indeed known that more than 90% of WM voxels contain complex geometries (14). As a consequence, the same potential wrong conclusions may be taken.

The above example puts the emphasis for the need of quantitative indices that should be able to take into account complex fiber geometries at the voxel level, and that be unbiased against such potential troubles. Different indices were considered in the past to overcome these issues, e.g., the Q-ball based generalized fraction anisotropy (18), or those based on Diffusion Kurtosis Imaging (55). As an alternative, multi-compartmental models were found to better describe non-gaussian water motion of in presence of complex geometries, like ball and stick (56), CHARMED (30), or NODDI (29). Those models however require high signal to noise ratios, a big number of diffusion directions, or multi-shell acquisitions.

### AFD interpretability at low b-values

AFD (25) was introduced to infer about intra-axonal volume fraction. Authors showed that radial DW signal is strictly linked to AFD which in turn is roughly proportional to intra-axonal volume fraction. In that way they found an average value of 75%, thus highlighting a close correspondence with an estimation of 80% for intra-cellular volume reported in Syková and Nicholson (26). However, such high correlation was reported to hold at high *b*-values (*b* = 3,000 s/mm^2^).

In this study we estimated, based on similar assumptions, an average value of 60% when considering voxels with high FA values (≥0.85) (Table 3). The reasons for such divergence may be manifolds and can be placed in two categories, namely biological and technical. From the technical point of view, a number of confounds may contribute to the underestimation intra-axonal volume fraction. First of all, in this study the estimation of the radial signal was based on DW volume whose diffusion gradient was the more transverse with respect to measured fODF peak. Due to the limited number of DW directions acquired, signal intensities could be chosen from a limited set of directions. It might be possible, therefore, that a slightly different spherical distribution could have contributed to ameliorate estimation of intra-axonal volume fraction. Moreover, the use of a single peak to detect underlying fiber direction is more error-prone in presence of noisy data, whereas an analogous search performed by integrating over fODF lobes may have led to better results. Eventually, due to partial volume effect, CSF component might influence our estimation by causing fictitious increasing of b0 signal and, consequently, a decrease of DW signal (26).

Another set of explanations is more inherently biological: it has been indeed recently found that the corpus callosum, a structure traditionally considered highly coherent in terms of fiber directionality, shows instead a rather elevated dispersion ranging from 10 to 35° (57). Therefore, the fiber dispersion might cause intra-axonal volume fraction underestimation. It is known that extra-cellular component decreases its contribute to diffusion weighted signal when high *b*-value are used (58); for lower *b*-values, like the one we used in the present study, extra-cellular compartment augments its influence on DW signal (59) and could potentially contribute to the observed underestimation.

Other micro-structures might further influence water fraction estimation due to water exchange between membrane barriers (60). It should be noted that in Syková and Nicholson (26) myelin component was considered part of the intra-cellular compartment. It has been shown that myelin water fraction ranges around 20% (61), and that the possible contemporary presence of both myelinated and unmyelinated axons might decrease the accuracy in myelin estimation (60).

Interestingly, if we were to add to myelin water component percentage the intra-axonal volume fraction as it was estimated in this study, we would almost equal the limit that Sykova and Nicholson measured for intra-cellular volume fraction (26). It could be however argued that myelin has a minor influence on our measure due to the short T2 decay of water trapped between myelin layers (61).

It is worth to mention that our results at *b* = 1,000 s/mm^2^ are in contraposition with numerical Monte-Carlo simulations provided in Raffelt et al. (25): there, it was reported that both restricted and permeable derived radial signals show a higher ratio between DW and unweighted signal (close to 90%) at low *b*-values. Such discrepancy could be likely due to some limitations in their model assumptions: as it was indeed reported there, possible relationships between diffusion phenomenon and other microbiological structures, e.g., myelin layers, may not have been properly modeled (25).

Pooling together all those considerations, we may conclude that, even when using a *b*-value of 1,000 s/mm^2^, and some limitations, we were still able to obtain a biologically meaningful estimation of intra-axonal volume fraction. As a consequence, we may be able to infer fiber population density even at such relatively low *b*-value. To the best of our knowledge, this is the first study exploring such implication.

### AFD and FA

We were particularly interested in showing the relationship between AFD and FA; it was already demonstrated (25, 27) that AFD provides richer insights on WM integrity when dealing with high demanding datasets.

Our aim was to show if the similar results could be confirmed with a low *b*-value (1,000 s/mm^2^) and a limited number of directions. Statistical analyses (Figure 4B) demonstrated that in almost all the cases, for all pathways of interest, a non-linear cubic S-shape relationship was detected (Figure 4C). As expected, for FA between 0.3 and 0.6, a linear relationship was observed; almost the same trend was found, for instance, when comparing FA with intra-axonal volume fraction provided by NODDI scheme (29). While a primary linear trend was evident, several points for which FA is relatively low (between 0.1 and 0.4) resulted to be coupled to AFD data increase. Such patterns were consistent for almost all subjects and correspond to voxels over which crossing or branching multiple fibers insist.

When investigating lower AFD values, we observed a relative high FA variability. Those points may likely correspond to situations in which multiple fibers have an angular distance so small to cause the tensor model to wrongly detect a single pathway pointing in a unique direction, therefore giving biased FA values. Such patterns caused the cubic S-shape fits we showed to better predict data in our subjects.

Angular resolution tends to overall decrease when fewer directions are used, and that could in turn cause inaccuracies in the correct estimation of complex fiber patterns even for fODF lobes. However, angular accuracy is by the way inherently more accurate when measured via CSD over DTI based estimation (19); therefore we can again conclude that AFD provides a more meaningful biological information.

## Conclusions

In accordance with existing literature, in this article we confirmed that CSD outperforms DTI one even in datasets with low demanding hardware settings (*b*-value = 1,000 s/mm^2^ and 30 diffusion encoding directions). Such setup needs to be preferred over more demanding ones especially when dealing with patients who are usually less collaborative due to their conditions.

Moreover, we showed that TB-RA of diffusion tensor parameters strongly depends on the cutoff chosen for voxels selection and on the method adopted for tractography, thus highlighting the importance of a careful check for principled data handling and analysis. Those considerations both apply to probabilistic DTI and CSD based tractographic analyses.

For the first time to our knowledge, we demonstrated that, with some limitations, AFD is a meaningful estimate of intra-axonal volume fraction when measured on diffusion acquisitions which can easily performed in clinical settings. Therefore, even with *b* = 1,000 s/mm^2^ and only 30 diffusion encoding directions, AFD can provide richer information than solely tensor features to investigate WM integrity in clinical studies.

To conclude, it is worth to notice that in this study a relatively small sample size has been adopted. In the future similar analyses on bigger populations will be therefore necessary to further confirm our results.

## Author contributions

ACal and AA contributed conception and design of the study, performed statistical analysis, interpreted data, and wrote the manuscript. EM contributed conception of the study and interpreted data. DM, ACac, SM, and GC revised the manuscript. MG and AQ contributed conception and design of the study and revised the manuscript. All authors read and approved the final manuscript.

### Conflict of interest statement

The authors declare that the research was conducted in the absence of any commercial or financial relationships that could be construed as a potential conflict of interest.
